# High-latitude ocean ventilation and its role in Earth's climate transitions

**DOI:** 10.1098/rsta.2016.0324

**Published:** 2017-08-07

**Authors:** Alberto C. Naveira Garabato, Graeme A. MacGilchrist, Peter J. Brown, D. Gwyn Evans, Andrew J. S. Meijers, Jan D. Zika

**Affiliations:** 1 Ocean and Earth Science, University of Southampton, National Oceanography Centre, Southampton, UK; 2 Department of Earth Sciences, University of Oxford, Oxford, UK; 3 National Oceanography Centre, Southampton, UK; 4 British Antarctic Survey, Cambridge, UK; 5 School of Mathematics and Statistics, University of New South Wales, Sydney, Australia

**Keywords:** ocean ventilation, Southern Ocean, Arctic Ocean, climate transitions

## Abstract

The processes regulating ocean ventilation at high latitudes are re-examined based on a range of observations spanning all scales of ocean circulation, from the centimetre scales of turbulence to the basin scales of gyres. It is argued that high-latitude ocean ventilation is controlled by mechanisms that differ in fundamental ways from those that set the overturning circulation. This is contrary to the assumption of broad equivalence between the two that is commonly adopted in interpreting the role of the high-latitude oceans in Earth's climate transitions. Illustrations of how recognizing this distinction may change our view of the ocean's role in the climate system are offered.

This article is part of the themed issue ‘Ocean ventilation and deoxygenation in a warming world’.

## 1. Introduction

Ocean ventilation is the process by which ‘young’ surface waters, which have recently been in contact with the atmosphere, are injected into the ocean interior and exported away from their sources [1,2]. The rate and distribution of ocean ventilation play an important role in Earth's climate through their regulatory influence on the oceanic uptake of heat and freshwater [3,4] and on many marine elemental cycles, such as those of oxygen and carbon [5,6]. The downward transport of surface waters propagates into the ocean interior the properties (e.g. temperature, salinity and concentrations of dissolved gases) imprinted by the atmosphere on the ocean surface in the areas of ventilation. In so doing, ventilation modulates the atmosphere–ocean partitioning of those properties [7,8], and shapes the pace of a range of climatically significant biogeochemical reactions (such as those regulating the extent of oxygen minimum zones [9]) in the ocean interior.

The high-latitude oceans (specifically, the Southern Ocean and the northern North Atlantic/Arctic Mediterranean) have long been recognized to provide the focus of global ocean ventilation [10,11], with a recent estimate suggesting that approximately two-thirds of the volume of the global ocean interior (and more than three-quarters of the global deep ocean below 1500 m) is ventilated at high latitudes [2]. The disproportionate importance of high-latitude regions in ventilating the ocean is commonly understood to arise from their hosting the downwelling limbs of the global overturning circulation [12,13]. These limbs are characterized by a geographically confined, net downward transport of mass between the surface and interior layers of the ocean, fed by nearby upwelling and diapycnal transformations (linked to air–ice–ocean interactions) of surface waters [12,14,15]. In this paradigm, the renewal of ocean interior properties is assumed to be fundamentally underpinned by high-latitude downwelling, and the role of ocean ventilation in Earth's climate is framed in those terms.

Illustrations of how this overturning-based view permeates discussions of the role of the ocean in Earth's major climate transitions abound in the recent literature. For example, the pronounced perturbations in atmospheric CO_2_ between the glacial and interglacial periods of the Quaternary have been attributed to changes in Southern Ocean ventilation [16], which are argued to stem from variations in the rate and structure of the overturning circulation in the region [17,18]. Similarly, the present role of high-latitude regions in the uptake of anthropogenic heat and carbon by the global deep ocean is rationalized in terms of the overturning circulation, whereby the bulk of the uptake must be effected by water masses downwelling at high latitudes [4,19].

In this article, we show that there are major elements of high-latitude ventilation and renewal of ocean interior properties that are controlled by processes occurring in *upwelling* layers, and that are thereby decoupled from the overturning circulation. In §2, we pursue this demonstration by revisiting the basic relationship between ventilation and overturning. A range of examples of how the two phenomena do in fact differ in the high-latitude oceans are provided in §3, and the climatic significance of these differences is illustrated in §4. Recommendations for the representation of these processes in conceptual models of the ocean's role in climate transitions are offered in §5.

## 2. De-coupling high-latitude ocean ventilation and overturning: theory

**Figure 1.**
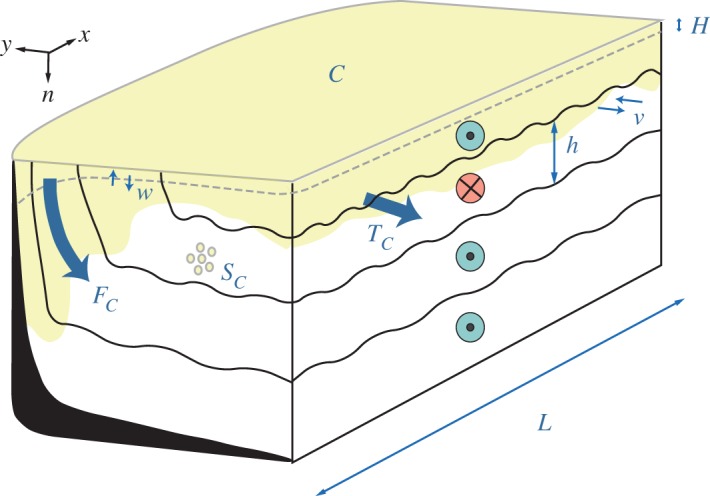
Schematic of ventilation in an idealized high-latitude ocean basin. The basin extends from a high-latitude landmass (or, equivalently, the North Pole) to an arbitrary mid-latitude zonal boundary of length *L*. The basin contains *n* sloping isopycnal layers of (spatially and temporally variable) thickness *h* and meridional velocity *v* that outcrop into the upper-ocean mixed layer (of depth *H*) within the region. Like the real-world high-latitude oceans, the basin hosts a double-celled overturning circulation, with inflow in intermediate layers and outflow in surface and deep layers [20,21]. Ventilation is quantified with a surface-sourced tracer *C*, which crosses the mixed layer base with vertical velocity *w* and at an integrated rate *F_C_*. The tracer has interior sources and sinks *S_C_* within the basin, and is exported through the mid-latitude boundary at a rate *T_C_*.

The relationship between high-latitude ocean ventilation and overturning can be readily illustrated by considering the idealized high-latitude ocean basin in figure 1. The basin extends from a high-latitude landmass (or, equivalently, the North Pole) to an arbitrary mid-latitude zonal boundary of length *L*, where this boundary is assumed to be zonally re-entrant or delimited by continents, for simplicity. Like real-world high-latitude oceans, the idealized basin contains a number of sloping isopycnal layers (indicated by variable *n*, and characterized by thickness *h*) that outcrop into the upper-ocean mixed layer (of depth *H*) within the region. Most generally, the circulation across the mid-latitude boundary of the basin (described by the meridional velocity, *v*) has horizontal, vertical and temporal structure, i.e. *v* = *v*(*x, n, t*), as does the density field along the boundary, i.e. *h* = *h*(*x, n, t*).

The rate of high-latitude ocean ventilation in this idealized basin at any given time may be quantified as the integrated transport (denoted as *F_C_*) of a tracer sourced in the atmosphere, *C*, across the mixed layer base within the basin,
2.1
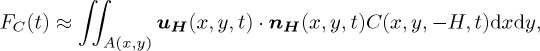

where ***u_H_*** is the three-dimensional velocity vector at the mixed layer base, ***n_H_*** is a unit vector normal to the mixed layer base, and the integral is taken over the area (*A)* of the mixed layer base within the basin. For a tracer with negligible sources or sinks in the basin's interior (such as chlorofluorocarbons or sulfur hexafluoride) in the limit of a stationary state, *F_C_* may be equivalently expressed as the integrated transport of the tracer across the basin's mid-latitude boundary (denoted as *T_C_*), i.e.
2.2
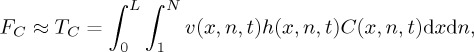

where *N* is the total number of isopycnal layers. If, instead, the tracer has potentially significant sources or sinks in the basin's interior (such as may be the case for, for example, oxygen or carbon), equation (2.2) becomes
2.3
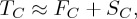

where *S_C_* denotes a source of tracer *C* in the basin's interior. Thus, the net influence of the high-latitude basin on the remainder of the ocean stems both from exchanges of tracer across the mixed layer base and from sources or sinks of tracer within the basin's interior. As high-latitude oceanic regions receive a net input of freshwater from precipitation, river runoff or ice sheet melt, *F_C_* and *T_C_* include a transport of tracer associated with the non-zero volume transport across the region's mixed layer base and mid-latitude boundary.

The integrated transport of the tracer across the basin's mid-latitude boundary, *T_C_*, may be partitioned into two distinct contributions by decomposing *v*, *h* and *C* in equation (2.3) into a zonal- and time-mean value along each isopycnal layer (indicated by 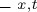) and perturbations from that value (denoted by primes), i.e.
2.4*a*
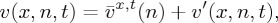

2.4*b*
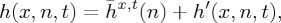

2.4*c*
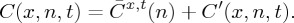

Substituting (2.4*a*)–(2.4*c*) into equation (2.2) yields
2.5
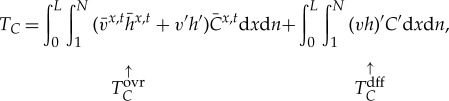

where terms involving solely one primed variable have been dropped, as they integrate to zero. The first term on the r.h.s. of equation (2.5), 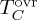, represents the advection of layer- and time-mean tracer concentrations by the overturning circulation, and is made up of mean (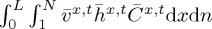) and eddy (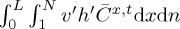) contributions. 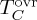 includes the transport of tracer associated with the non-zero volume transport across the basin's mid-latitude boundary. The second term on the r.h.s. of equation (2.5), 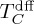, represents the meridional transport of tracer associated with perturbations in the meridional thickness transport (*vh*) and in the tracer concentration relative to layer- and time-mean values of those quantities. This term is often referred to as ‘eddy diffusion’ in the oceanographic literature, and may be parametrized through a downgradient diffusive closure [22,23]. Note that, in this general formulation, the term ‘eddy’ refers exclusively to perturbations from layer-wise, temporal means, and carries no connotations as to the dynamical nature of those perturbations (i.e. ‘eddy’ perturbations may be associated with transient mesoscale eddies, quasi-stationary large-scale gyres, or re-circulations of any arbitrary lateral and temporal scales).

Equation (2.5) makes explicit the relationship between high-latitude ocean ventilation, measured by *T_C_* as per equation (2.3), and the contribution of the overturning circulation, 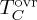. The overturning-based paradigm of high-latitude ocean ventilation that commonly underpins discussions of the ocean's role in climate assumes that 
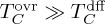
, giving
2.6
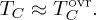


The extensive invalidity of this approximation, and thereby the overturning-based view of high-latitude ocean ventilation, is illustrated with observations in the next section.

## 3. De-coupling high-latitude ocean ventilation and overturning: observations

Observational evidence of a de-coupling between high-latitude ocean ventilation and overturning may be readily obtained from inspection of the meridional transport of a suite of tracers sourced at the surface of the high-latitude oceans. In this section, we provide three significant examples from the recent oceanographic literature, but a range of other illustrations may be garnered from older work.

### (a) The Antarctic Circumpolar Current

The first example pertains to the injection of surface waters (which have been renewed by exchanges with the atmosphere) into the interior layers of the Antarctic Circumpolar Current (ACC) system. The prevalent view of ocean ventilation in the region [24,25] presumes that surface waters enter the mid-latitude pycnocline primarily near the ACC's northern edge, where formation, downwelling and equatorward export of Antarctic Intermediate Water (AAIW) and Subantarctic Mode Water (SAMW) occur in association with a convergence of the meridional overturning flow in the upper ocean. Thus, in this paradigm, tracers sourced at the surface are expected to penetrate the interior of the ACC along isopycnal layers embedded within those downwelling water masses, and to be scarce or absent in upwelling layers (contained primarily in Circumpolar Deep Water, CDW)—particularly if the tracers have significant deep-ocean interior sinks.

To assess whether this view holds in the ocean, we examine the distribution of a direct metric of the rate of along-isopycnal injection of surface-sourced tracers across a sector of the ACC, and contrast it with the meridional overturning circulation in the same region. To introduce this metric, it is useful to recall that the large-scale temperature–salinity structure of the ocean is established as surface water properties are transferred into the interior by the large-scale, slowly evolving ocean circulation (linked to 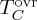 in equation (2.5)) and by fluctuating, turbulent flows acting on a wide range of spatial and temporal scales (linked to 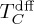 in equation (2.5)). As shown by [26], this may be expressed as
3.1
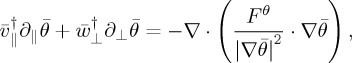

where *θ* is potential temperature; overbars and asterisks respectively refer to a slowly changing mean state and fluctuations; 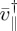 and 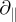 are the meridional components of the residual-mean (i.e. overturning) velocity [27] and the gradient operator (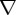) directed along isopycnals; 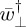 and 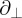 are the diapycnal components of the residual-mean velocity and the gradient operator; 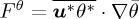 is the rate of production of temperature variance by turbulent flows (often referred to as ‘mixing’) with velocity ***u***; and stationarity and zonal homogeneity have been assumed, as is arguably appropriate to the ACC. *F^θ^* can be decomposed into isopycnal and diapycnal components, as 
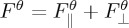
. It can be shown [28,29] that the turbulent isopycnal production of temperature variance (also known as isopycnal mixing), 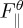, is commonly associated with mesoscale eddies stirring the large-scale temperature gradient along isopycnals, whereas the turbulent diapycnal production of temperature variance (diapycnal mixing), 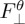, is induced by small-scale turbulence (generated by, for example, breaking internal waves) stirring the large-scale temperature gradient across isopycnals.

Equation (3.1) states that the temperature of the ACC interior is set by advection following the overturning circulation and by turbulent (isopycnal and diapycnal) mixing. As ventilation brings into contact surface and interior waters with contrasting properties, a hallmark signature of ventilation [26,28] is the generation of significant large-scale temperature gradients in the ocean interior that mesoscale eddies subsequently stir into filaments of O(1 km) horizontal scale, leading to a prominent enhancement of 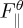 relative to 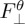, i.e. 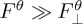. These filaments are eroded away by diapycnal mixing on a time scale of O(1 year) [26,29]. As a result, regions of the ocean interior that have not been ventilated on that time scale are characterized by weak large-scale temperature gradients and correspondingly negligible isopycnal mixing, yielding 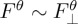 there. Thus, the recentness of ventilation at any point in the ACC interior can be quantified unambiguously from the ratio 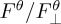.

**Figure 2.**
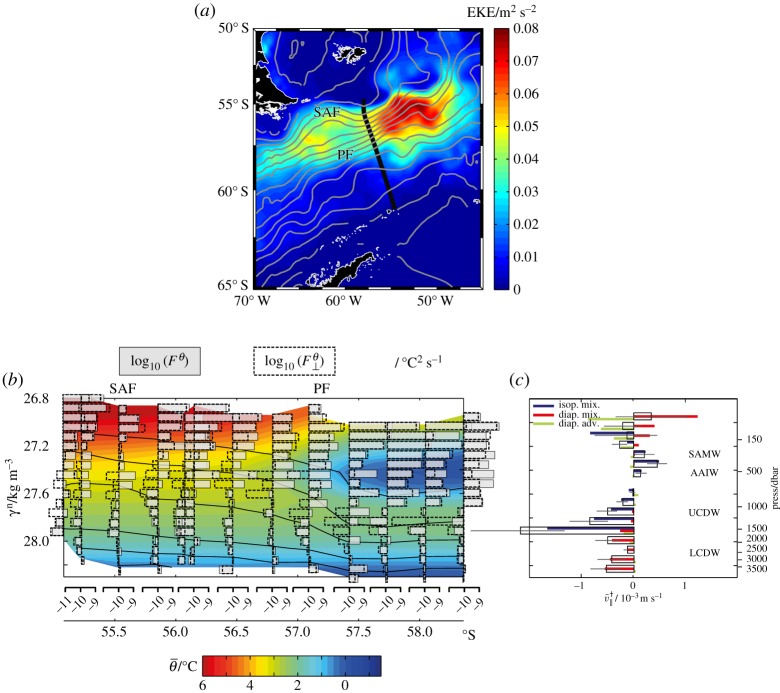
(*a*) Position of the oceanic microstructure section discussed in §3a (in black), superimposed on mesoscale eddy kinetic energy calculated from AVISO gridded altimetry (colour) and mean dynamic topography at intervals of 0.1 m (in grey). The two main fronts of the ACC are labelled: PF (Polar Front), SAF (Subantarctic Front). (*b*) Meridional section of log_10_*F^θ^* (grey bars) and 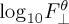 (dashed bars) estimated from microstructure measurements by [26], with 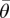 shown by background shading. Pressure contours of 150, 500, 1000, 2000, 3000 and 4000 dbar are overlaid in black. ACC frontal positions are indicated on the upper axis. (*c*) Section-characteristic profile of 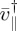 (open bars, with error bars), with contributions to 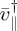 balanced by the terms involving 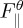 (isopycnal mixing), 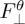 (diapycnal mixing) and 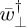 (diapycnal advection) in equation (3.1) shown by blue, red and green bars, respectively. The section-mean pressure profile of density surfaces is indicated on the right-hand axis. Adapted from [26].

A quasi-meridional section of *F^θ^* and 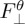 across the southwest Atlantic sector of the ACC (figure 2*a*) is shown in figure 2*b*, alongside an estimate of the section-mean meridional-isopycnal velocity, 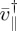, describing the overturning circulation in figure 2*c*. Both of these variables are diagnosed from oceanic microstructure measurements following the methodology described in [26], whereby 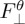 and *F^θ^* are respectively estimated from shear and temperature microstructure data. The validity of the assumption of zonal homogeneity implicit in the calculation is also discussed by those authors. The overturning circulation exhibits the characteristic southward/upward flow of CDW (*γ*^n^ > 27.5 kg m^−3^, where *γ*^n^ is the neutral density of [30]) and northward/downward flow of AAIW and SAMW (27.2 < *γ*^n^ < 27.5 kg m^−3^), with insignificant flow in the lightest density classes. The ratio 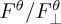 is generally large (of order 10–100) equatorward of the Polar Front, where mode and intermediate waters outcrop into the winter mixed layer (approximated by the 150 m depth contour in figure 2*b*). This is as expected from an overturning-based view of ventilation, whereby distinctively cold and fresh waters are injected into the ocean interior at the outcrops, and transported by the overturning circulation northward and downward into the mid-latitude pycnocline. Similarly, the prevalence of 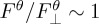 in the Lower CDW classes (*γ*^n^ > 28.0 kg m^−3^), which suggests that those waters have not experienced recent ventilation, also conforms to expectations from an overturning-based paradigm of ventilation. This is because meridional flow in those layers is directed toward (rather than away from) their closest outcropping sites, found well to the south of the ACC, such that the overturning circulation opposes (rather than promotes) the northward and downward penetration of surface waters along isopycnals.

In contrast, a pronounced qualitative departure from the overturning-based view of ventilation occurs in the Upper CDW classes (27.5 < *γ*^n^ < 28.0 kg m^−3^). There, 
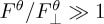
 and southward/upward flow occur concurrently, indicating that those layers are being actively ventilated by surface waters against the action of advection by the overturning circulation. This feature may be interpreted to result from isopycnal mixing by the ACC's energetic mesoscale eddy field driving a northward/downward transport of surface waters, associated with in-phase, eddy-induced fluctuations in the meridional thickness transport and in temperature relative to the local mean values of those quantities [22,23]. This is equivalent to the ‘eddy diffusion’ characterized in equation (2.5), implying that 
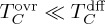
 in the Upper CDW.

To conclude, an overview of the validity of the overturning-based paradigm of ventilation in the ACC is provided by figure 2*c*. The paradigm holds qualitatively in northward/downward- (southward/upward-) flowing layers where isopycnal (diapycnal) mixing balances the overturning circulation across the ACC's mean temperature structure (i.e. in SAMW/AAIW and Lower CDW), yet fails in southward/upward-flowing layers where isopycnal mixing balances the overturning circulation (i.e. in Upper CDW). In §4, we will argue that this failure is indicative of a widespread quantitative breakdown of the paradigm in the ACC.

### (b) The polar Southern Ocean

**Figure 3.**
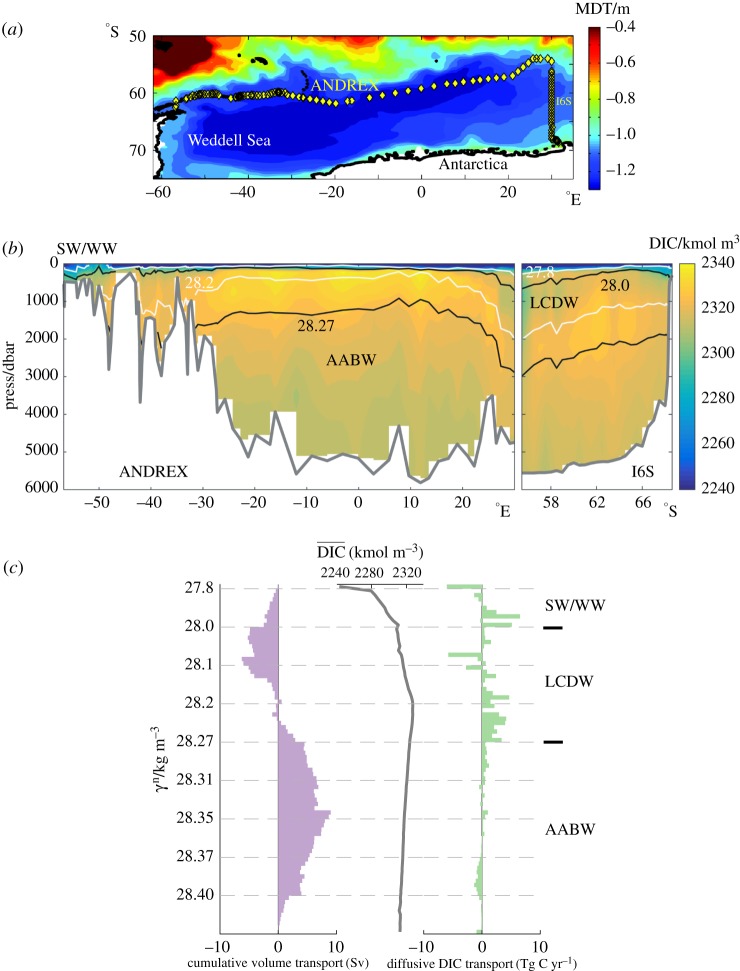
(*a*) Position of the oceanographic transects used in defining the carbon budget of the Weddell Gyre discussed in §3b (yellow diamonds), superimposed on the mean dynamic topography (colour) from the Southern Ocean State Estimate [31] in 2005. (*b*) Dissolved inorganic carbon (DIC) along the transects in (*a*) (colour). Isopycnal surfaces bounding the major water masses of the region (SW = Surface Water; WW = Winter Water) are indicated in black, and those delimiting the two overturning circulation cells (in (*c*)) are shown in white. (*c*) Volume transport across the rim of the Weddell Gyre accumulated from the bottom (defined as positive when directed northward/equatorward), mean DIC along the transects in (*a*), and diffusive DIC transport out of the Weddell Gyre, displayed as a function of neutral density. The major water masses are indicated on the right-hand axis. Adapted from [32,33].

The second illustration of the significant de-coupling between high-latitude ocean ventilation and overturning entails the budget of a surface-sourced, biologically active tracer (carbon) in the Weddell Gyre, which spans over one quarter of the polar Southern Ocean (figure 3*a*). A common view of ocean ventilation in the region [12,20] emphasizes the injection of surface waters to the deep ocean via the formation and sinking of Antarctic Bottom Water (AABW) near the Antarctic margins, sustained by the densification of Lower CDW and upper-ocean waters forced by local atmospheric cooling and sea ice production [32]. Comparatively weaker upwelling of Lower CDW into lighter upper-ocean layers occurs further offshore [20], and is presumed to be of little significance to ventilation. Since carbon is sourced at the surface and enriched in the ocean interior (through remineralisation processes), it is more abundant in the upwelling, ‘old’ Lower CDW than in the downwelling, recently ventilated AABW and near-surface waters of the Weddell Gyre (figure 3*b*,*c*). Thus, in an overturning-based paradigm of ventilation, the region would be expected to act as a net *source* of carbon to the atmosphere.

The inapplicability of this view in the Weddell Gyre is succinctly expressed by the net carbon budget of the region synthesized in table 1. See [33,34] for a complete description of the carbon budget calculation, which does not assume the carbon system to be in a stationary state. The Weddell Gyre is an important net *sink* of carbon from the atmosphere of 44 ± 10 Tg C yr^−1^. Although the overturning component of the transport (i.e. 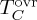) across the gyre's rim imports −2 ± 10 Tg C yr^−1^ to the region, it is overwhelmed by an oppositely-signed diffusive component of the transport (i.e. 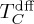), which exports 46 ± 10 Tg C yr^−1^ from the region. As illustrated by figure 3*c*, the oceanic uptake of carbon in the Weddell Gyre encapsulated in 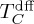 stems primarily from the enrichment in carbon of Lower CDW recirculating around the gyre. Such enrichment is driven by an interior source: the remineralization of sinking organic matter formed by phytoplankton during photosynthesis in the surface waters of the central gyre [33].

**Table 1.** Transport of dissolved inorganic carbon across the rim of the Weddell Gyre, decomposed into overturning and diffusive components, and into major water masses. Positive values represent a sink of carbon from the atmosphere.

layer (density class, kg m^−3^)	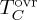 (Tg C yr^−1^)	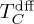 (Tg C yr^−1^)	*T_C_* (Tg C yr^−1^)
Surface and Winter Waters (*γ*^n^ < 27.8)	4271	13	4284
Lower CDW (27.8 < *γ*^n^ < 28.27)	−8206	34	−8172
AABW (*γ*^n^ > 28.27)	3933	−1	3932
Full depth	−2 ± 10	46 ± 10	44 ± 10

To summarize, the overturning-based paradigm of ventilation fails to adequately characterize the Weddell Gyre carbon sink because 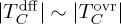 there. Although this conclusion is specific to carbon, it is likely to hold for a wide range of biologically active tracers of air–sea gas exchange (such as oxygen) that are coupled to carbon by biogeochemical stoichiometries [35]. A further example of the comparable significance of 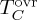 and 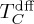 in the polar Southern Ocean is offered by an anthropogenic, biologically inert tracer (chlorofluorocarbon-11), which has been shown to be injected at comparable rates into the AABW and Lower CDW layers by shelf waters cascading down the Antarctic continental slope [36].

### (c) The Arctic Ocean

**Figure 4.**
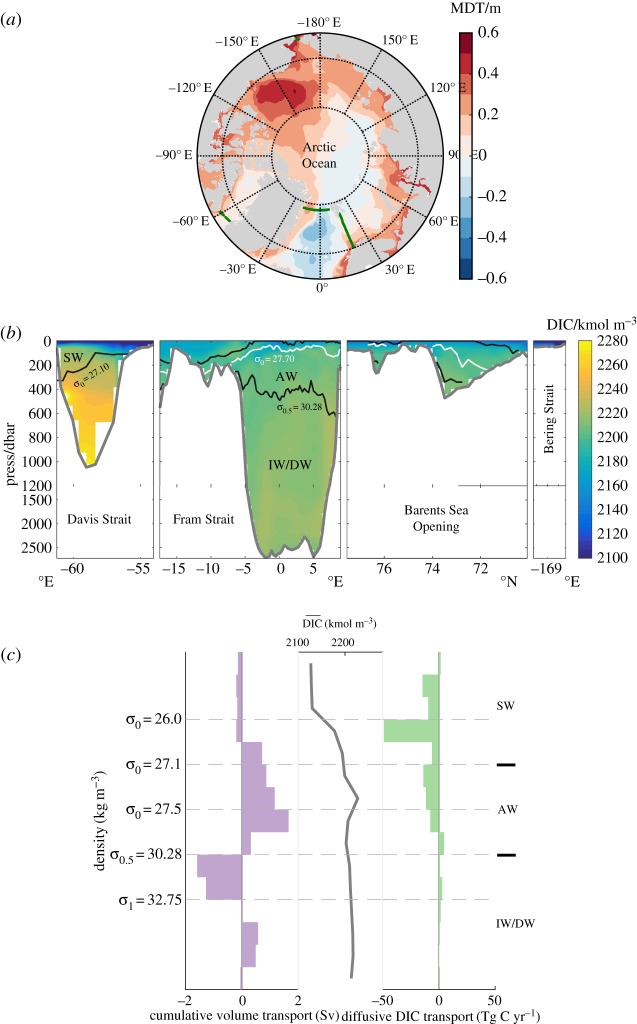
(*a*) Position of the oceanographic transects used in defining the carbon budget of the Arctic Ocean discussed in §3c (green lines), superimposed on the mean dynamic topography (shading) from the ORCA12 model [37] in 2008. (*b*) Dissolved inorganic carbon (DIC) along the transects in (*a*) (colour). Isopycnal surfaces bounding the major water masses of the region (SW = Surface and Subsurface Waters; IW/DW = Intermediate and Deep Waters) are indicated in black, and those delimiting the two overturning circulation cells (in (*c*)) are shown in white. (*c*) Volume transport across the rim of the Arctic Ocean accumulated from the bottom (defined as positive when directed northward/poleward), mean DIC along the transects in (*a*), and diffusive DIC transport into the Arctic Ocean, displayed as a function of neutral density. The major water masses are indicated on the right-hand axis. Adapted from [38].

The uptake of carbon by the Arctic Ocean (figure 4*a*) provides the final illustration of the general invalidity of the overturning-based paradigm of high-latitude ocean ventilation. As in the Weddell Gyre, a common view of ventilation in the Arctic [21,39] highlights the injection of surface waters to the ocean interior via a double overturning cell, wherein a mid-depth water mass (commonly termed Atlantic Water, AW) is transformed into lighter upper-ocean waters and denser Arctic Intermediate and Deep Waters. Greater freshwater runoff from neighbouring landmasses results in the overturning circulation in the Arctic Ocean being significantly weaker than that in the Weddell Gyre. As in the latter case, carbon is commonly more abundant in the inflowing mid-depth layer (i.e. in AW) than in overlying waters (figure 4*b*,*c*), although Arctic Intermediate and Deep Waters are characterized by comparably high carbon concentrations to those in AW. Thus, an overturning-based view of ventilation would suggest that the Arctic's upper layers should act as a net *source* of carbon to the atmosphere, and present an ambiguous picture of the contribution of the region's deeper layers.

The net carbon budget of the Arctic Ocean is synthesized in table 2. See [38] for a complete description of the carbon budget calculation. Contrary to expectations from the overturning-based view, the Arctic Ocean is an important net *sink* of carbon from the atmosphere of −214 ± 49 Tg C yr^−1^. This equatorward transport of carbon is approximately equipartitioned between its overturning (−115 ± 49 Tg C yr^−1^, which is fully accounted for by the transport of carbon associated with the net oceanic mass flux out of the Arctic) and diffusive (−99 ± 49 Tg C yr^−1^) components. Similarly to the Weddell Gyre, the diffusive contribution arises from the enrichment in carbon of mid-depth and upper-ocean waters recirculating around the Arctic basin (figure 4*c*). As in the Weddell case, this enrichment is primarily driven by an interior source associated with the remineralization of sinking organic matter, focused directly to the north of Davis Strait [38].

**Table 2.** Transport of dissolved inorganic carbon across the rim of the Arctic Ocean, decomposed into overturning and diffusive components, and into major water masses. Positive values represent a source of carbon to the atmosphere.

layer (density class, kg m^−3^)	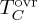 (Tg C yr^−1^)	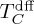 (Tg C yr^−1^)	*T_C_* (Tg C yr^−1^)
Surface and Subsurface Waters (*σ*_0_ < 27.1)	−832	−77	−910
Atlantic Water (*σ*_0_ > 27.1, *σ*_0.5_ < 30.28)	2042	−28	2014
Intermediate and Deep Waters (*σ*_0.5_ > 30.28)	−1325	6	−1319
Full depth	−115 ± 49	−99 ± 49	−214 ± 49

In conclusion, the operation of the Arctic Ocean carbon sink does not conform to the overturning-based paradigm of ventilation. Although the overturning circulation contributes significantly to the uptake of carbon by the region, the magnitude of the uptake is significantly underestimated by the neglect of the diffusive component, i.e. 
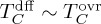
. As argued in §3b, this conclusion is likely extrapolable to a wide range of biologically active tracers of air–sea gas exchange, such as oxygen.

## 4. Climate impacts of high-latitude ocean ventilation

The range of illustrations offered in §3 attests to a significant de-coupling between high-latitude ocean ventilation and overturning. This distinction is likely relevant not only to the ocean's modern quasi-equilibrium state, but also to its transient behaviour during major climate transitions. To illustrate this point, we next outline recent evidence suggesting that contemporary oceanic climate change projects strongly onto elements of high-latitude ventilation unrelated to overturning. We restrict this review to the preceding case examples for the sake of succinctness.

**Figure 5.**
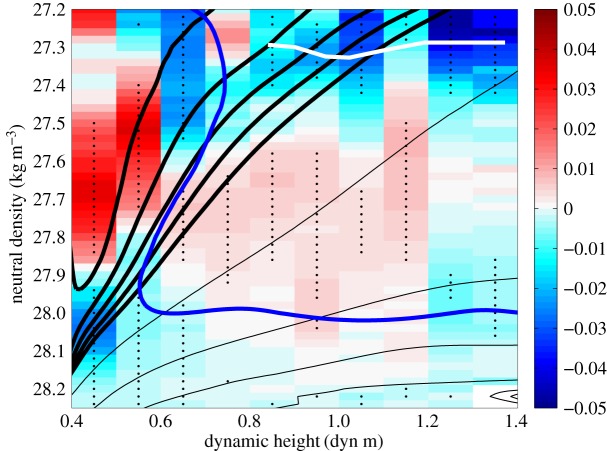
Circumpolar temperature trend (in °C yr^−1^) across the ACC between 1984 and 2012, calculated from ship-based hydrographic observations [40]. The dynamic height displayed along the *x*-axis is computed between 400 and 2000 dbar. Bold black contours indicate mean pressure surfaces at 100 dbar intervals, in the 100–500 dbar range. Fine black contours show mean pressure surfaces at 1000 dbar intervals, in the 1000–4000 dbar range. Stippling indicates trends that are statistically significant with 95% confidence. The white line shows the position of the mean salinity minimum, indicative of AAIW; and the blue line shows the location of the 2°C isotherm, the vertical segment of which marks the northern terminus of the Polar Front. The Subantarctic Front is located at the right-hand boundary of the figure.

A clear illustration of the significance of ventilation of upwelling layers in contemporary climate change is provided by the oceanic heat uptake in the ACC in recent decades. Observations of temperature change in the ACC (figure 5) reveal a warming of the region's deep layers by up to 0.015 K yr^−1^ focused in the density class of the upwelling Upper CDW. The warming occurs around and across the Southern Ocean and is most pronounced near the surface, suggesting that the increase in temperature may be driven by oceanic heat gain from the atmosphere [40,41]. This interpretation is compatible with a recent idealized, eddy-resolving modelling study of the sensitivity of ventilation in the ACC to wind forcing perturbations [42], which shows that variations in ventilation on interannual to decadal time scales are primarily associated with changes in the diffusive action of mesoscale eddies (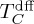) rather than changes in overturning (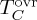).

**Figure 6.**
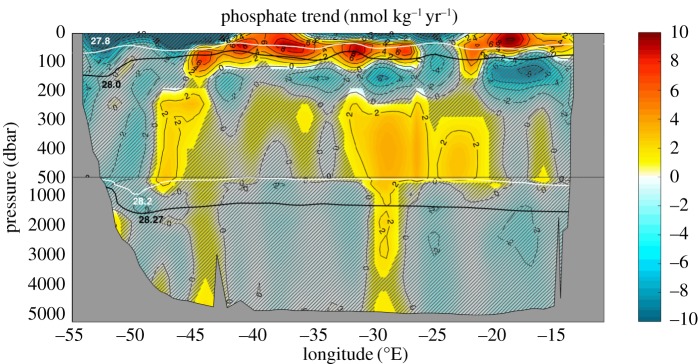
Rate of change in dissolved phosphate concentration along a repeat transect crossing the Weddell Gyre between 1996 and 2011. Hatching indicates trends that are not statistically significant with 95% confidence. Neutral density surfaces bounding the major water masses of the region are indicated in black, and those delimiting the two overturning circulation cells are shown in white (figure 3*c*). Adapted from [43].

Indications of the impact of contemporary changes in the carbon budgets of the Weddell Gyre and Arctic Ocean on the diffusive component of regional carbon export may be found in repeat biogeochemical observations recently reported in the literature. Although those observations do not always include dissolved inorganic carbon, changes in carbon may be inferred from measured variations in nutrient concentrations, assuming Redfield stoichiometry to hold to a good approximation [35]. In the Weddell Gyre, a significant enrichment in nutrients [43] and carbon [44] has occurred over the last two decades (figure 6) in precisely the layers (Lower CDW and overlying pycnocline waters) in which the diffusive transport of carbon is concentrated (figure 3*c*). Similarly, in the Arctic Ocean, available evidence indicates that the most pronounced biogeochemical shifts of the last two decades have been focused in Baffin Bay [45], where the biologically mediated carbon enrichment of mid-depth and upper-ocean waters occurs that underpins the diffusive component of regional carbon export (figure 4*c*).

There are no estimates at present of how these changes in the high-latitude oceans are impacting their role in renewing ocean interior properties. However, the observed spatial configuration of the changes is consistent with that impact being mediated significantly by the diffusive component of ocean ventilation.

## 5. Concluding remarks

In this article, we have used an idealized theoretical model and a range of observations to show that high-latitude ventilation and renewal of ocean interior properties are significantly decoupled from the overturning circulation. On this basis, we argue that the commonly invoked, overturning-based paradigm of high-latitude ocean ventilation (which implicitly assumes that exchanges of ventilation tracers between the high- and mid-latitude oceans are dominated by the overturning circulation) does not provide a widely valid conceptual framework for interpreting the role of the high-latitude oceans in Earth's climate transitions. This is because horizontal re-circulations of a range of lateral and temporal scales, such as transient mesoscale eddies and basin-scale gyres, are major players in the transfer of ventilation tracers between the high- and mid-latitude oceans. The transport effected by these re-circulating flows is often referred to as ‘eddy diffusion’ in the oceanographic literature [22].

We propose that the simplest, most likely appropriate paradigm of high-latitude ocean ventilation should be based on the assertion that the dominant balance of overturning and diffusive components of ventilation in the present climate is maintained in a perturbed climatic state. There are two basic requirements to this paradigm's application to assessing or conceptually modelling how variations in ventilation shape the evolving distribution of a tracer of air–sea gas exchange. First, the relative importance of overturning and diffusive contributions for that tracer must be quantified at the outset of the experiment. Second, a mechanistically based representation of the diffusive component must be defined in terms of the properties of the pertinent horizontal re-circulations and large-scale tracer field. For example, if one were to apply this approach to the illustrative problems introduced in §1, the role of changes in the horizontal circulation and biological pump of Southern Ocean polar gyres would need to be acknowledged and parametrized in investigating the causes of glacial–interglacial fluctuations in atmospheric CO_2_. Similarly, changes in the diffusive action of mesoscale eddies [42] would have to be factored into the on-going discussion of how atmospheric forcing of the Southern Ocean regulates the uptake of anthropogenic heat and carbon by the global deep ocean. We thus suggest that significant new insights into the climatic impacts of high-latitude ocean ventilation are likely to arise from the application of this alternative paradigm.

## Data accessibility

The data used in constructing figures 2–4 are available from the British Oceanographic Data Centre (http://www.bodc.ac.uk). The data entering figures 5–6 can be found in the CLIVAR & Carbon Hydrographic Data Office (CCHDO; http://cchdo.ucsd.edu).
